# PI3K/Akt/mTOR pathway inhibitors enhance radiosensitivity in radioresistant prostate cancer cells through inducing apoptosis, reducing autophagy, suppressing NHEJ and HR repair pathways

**DOI:** 10.1038/cddis.2014.415

**Published:** 2014-10-02

**Authors:** L Chang, P H Graham, J Hao, J Ni, J Bucci, P J Cozzi, J H Kearsley, Y Li

**Affiliations:** 1 Cancer Care Centre and Prostate Cancer Institute, St. George Hospital, SESLHD, Kogarah, New South Wales 2217, Australia; 2 Faculty of Medicine, St. George and Sutherland Clinical School, University of New South Wales, Kensington, New South Wales 2052, Australia; 3 Department of Surgery, St. George Hospital, Kogarah, New South Wales 2217, Australia

## Abstract

The PI3K/Akt/mTOR pathway has a central role in cancer metastasis and radiotherapy. To develop effective therapeutics to improve radiosensitivity, understanding the possible pathways of radioresistance involved and the effects of a combination of the PI3K/Akt/mTOR inhibitors with radiotherapy on prostate cancer (CaP) radioresistant cells is needed. We found that compared with parent CaP cells, CaP-radioresistant cells demonstrated G0/G1 and S phase arrest, activation of cell cycle check point, autophagy and DNA repair pathway proteins, and inactivation of apoptotic proteins. We also demonstrated that compared with combination of single PI3K or mTOR inhibitors (BKM120 or Rapamycin) and radiation, low-dose of dual PI3K/mTOR inhibitors (BEZ235 or PI103) combined with radiation greatly improved treatment efficacy by repressing colony formation, inducing more apoptosis, leading to the arrest of the G2/M phase, increased double-strand break levels and less inactivation of cell cycle check point, autophagy and non-homologous end joining (NHEJ)/homologous recombination (HR) repair pathway proteins in CaP-radioresistant cells. This study describes the possible pathways associated with CaP radioresistance and demonstrates the putative mechanisms of the radiosensitization effect in CaP-resistant cells in the combination treatment. The findings from this study suggest that the combination of dual PI3K/Akt/mTOR inhibitors (BEZ235 or PI103) with radiotherapy is a promising modality for the treatment of CaP to overcome radioresistance.

Radiotherapy (RT) is an important treatment option for prostate cancer (CaP) patients detected at early-stage or advanced-stage disease. Despite appropriate RT, up to 30% of treated high-risk CaP patients often experience local relapse and progression to metastatic disease.^1^ One main reason for these failures following RT is because of radioresistance of a subpopulation of CaP clones within tumor. Therefore, radioresistance is a major challenge for the current CaP RT. RT dose escalation techniques have been used to counteract radioresistance. However, further dose escalations to 82 Gy in a phase II trial yielded significant acute and late morbidity.^2^ Although three-dimensional conformal RT, intensity-modulated radiation therapy and image guided radiation therapy can increase the dose to local CaP and improve control rate,^3^ the clinical outcomes indicate that these advanced approaches cannot completely overcome radioresistance in CaP.^4^ Thus, modalities for improving the therapeutic efficacy of RT for locally confined or locally advanced CaP are warranted to increase sensitivity of radiation treatment in optimizing radiation effect and minimizing radioresistance influence.

The PI3K/Akt/mTOR pathway is an important intracellular signaling pathway in regulating cell growth, survival, adhesion and migration, particularly during cancer progression, metastasis and radioresistance,^5, 6, 7, 8^ and is frequently activated in cancer cells. PI3K activates a number of downstream targets including the serine/threonine kinase Akt that activates mTOR. Many valuable inhibitors targeting one protein (single inhibitor) or two proteins at the same time (dual inhibitor) in the pathway have been developed in recent years.

BKM120 is a single PI3K inhibitor by inhibiting p110*α/β/δ/γ* and often results in tumor suppression,^9^ and Rapamycin is a single mTOR inhibitor and has been used in clinical trials against various cancer types.^10^ NVP-BEZ235 (BEZ235) is a potent dual pan-class I PI3K and mTOR inhibitor that inhibits PI3K and mTOR kinase activity and has been used in preclinical studies in many cancers to demonstrate excellent anticancer effects.^11^ In addition, this inhibitor was the first PI3K/mTOR dual inhibitor to enter clinical trials in 2006.^12^ PI103 is another potent dual pan-class I PI3K and mTOR inhibitor and selectively targets DNA-PK, PI3K (p110*α*) and mTOR.^13^ No reports have been published to test them in CaP-radioresistant (RR) cells as radiosensitizers to improve radiosensitivity so far. The mechanisms of these inhibitors in combination with RT in the treatment of CaP are unclear.

Under a low-dose radiation treatment, we have recently developed three CaP-RR cell lines with increased colony formation, invasion ability, sphere formation capability and enhanced epithelial–mesenchymal transition (EMT) and cancer stem cell (CSC) phenotypes and the activation of the PI3K/Akt/mTOR signaling pathway.^7^ In addition, we also found that the PI3K/Akt/mTOR pathway is closely linked with EMT and CSCs.^7^ Therefore, these CaP-RR cells, representative of the source of CaP recurrence after RT, may provide a very good model to mimic a clinical radioresistance condition as well as to examine the efficacy of these single and dual PI3K/Akt/mTOR inhibitors for their radiosensitization effects.

Here, we investigated (1) whether cell cycle distribution, cell cycle check point proteins, apoptosis, autophagy and DNA repair pathways are involved in CaP radioresistance; (2) the link between radiosensitization effects and cell cycle distribution after treatment with a combination of dual inhibitors (BEZ235 and PI103) and single inhibitors (BKM120 and Rapamycin) with RT in CaP-RR cells *in vitro*; (3) whether cell death pathways (apoptosis and autophagy), DNA repair pathways (non-homologous end joining (NHEJ) and homologous recombination (HR)) are associated with CaP radiosensitivity after treatment with combination of dual or single inhibitors with RT.

## Results

### Cell cycle distribution and checkpoint protein changes in CaP-RR cells

**Figure 1:**
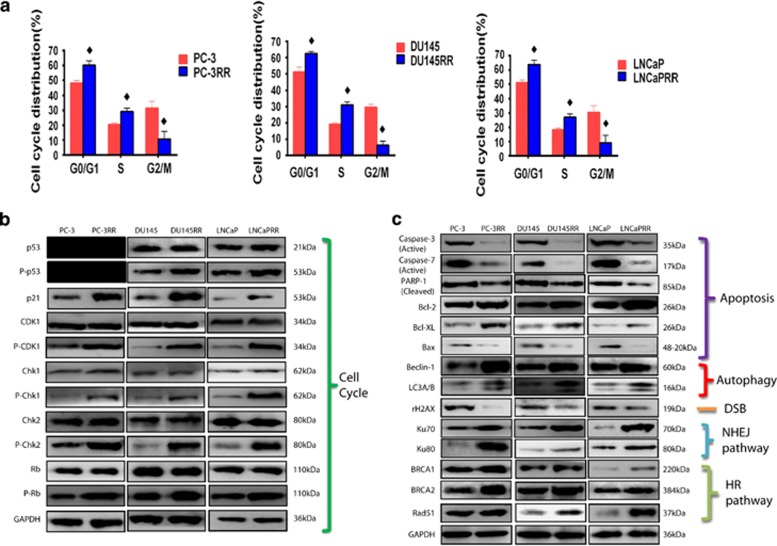
CaP-RR cells induce cell cycle redistribution, reduce apoptosis pathway proteins, increase autophagy, and NHEJ and HR pathway proteins. (**a**) Cell cycle distributions were analyzed with flow cytometry and significant difference was found in G0/G1, S and G2/M phases between CaP-RR and CaP cells (♦*P*<0.05). (**b**) Cell cycle-related proteins (p53, p-p53, p21, CDK1, p-CDK1, Chk1, p-Chk1, Chk2, p-Chk2, Rb and p-Rb) were determined using western blot analysis and phosphosed proteins (p-p53, p-p21, CDK1, p-Chk1, p-Chk2 and p-Rb) were increased (activated) in CaP-RR cells. (**c**) Apoptosis proteins (active caspase-3, active caspase-7, cleaved PARP-1 and Bax) and DSB marker (*γ*H2AX) were reduced, and anti-apoptosis (Bcl-2 and Bcl-xl), autophagy (Beclin-1 and LC3A/B), NHEJ (Ku70 and Ku80) pathway, HR pathway (BRCA1, BRCA2 and RAD51)-related proteins were increased (activated) in CaP-RR cells. GAPDH was used as a loading control. Typical images are shown from three independent experiments (*n*=3). CaP: prostate cancer; DSB: double strand break; p: phosphor; RR: radioresistant

The percentage of G0/G1 and S cell populations was significantly increased, whereas the percentage of G2/M cell population was obviously reduced in CaP-RR cells compared with CaP cells in three CaP cell lines (PC-3, DU145 and LNCaP; *P*<0.05; Figure 1a); however, the degree of the cell increase in S population in CaP-RR cells was much less than that in G0/G1 population in CaP cells, suggesting that more CaP-RR cells were arrested in G0/G1 and S phases, and that radioresistance triggered a significant reduction of G2/M arrest accompanied with an increase in G0/G1 and S portions (Figure 1a). The details of the difference of cell cycle distributions in CaP-RR and CaP cell lines are summarized in Supplementary Table S1.

As a cell cycle and proliferation marker, the expression of Ki67 was significantly reduced in CaP-RR cells compared with CaP cells (data not shown). P53 is negative in PC-3 cells and the expression of both phosphor-p53 (p-p53) and p21 was found to be increased, whereas no difference was found in the expression of p53 in CaP-RR cells compared with CaP cells (Figure 1b), indicating that the p53–p21 axis is activated in CaP-RR cells. The expression of p-CDK1, p-Chk1, p-Chk2 and p-Rb proteins was found to be increased in CaP-RR cells compared with that in CaP cells, whereas no change was found in the expression of CDK-1, Chk1, Chk2 and Rb between CaP-RR and CaP cells (Supplementary Figure S1 and Supplementary Table S2), indicating that cell cycle checkpoint proteins (p-CDK1, p-Chk1, p-Chk2 and p-Rb) are activated in CaP-RR cells (Figure 1b).

### CaP radioresistance inhibits the apoptosis pathway, activates autophagy and both NHEJ and HR DNA repair pathways

The expression of the active caspase-3, active caspase-7, cleaved PARP-1 and Bax proteins was obviously reduced, whereas the expression of Bcl-2 and Bcl-xl proteins was increased in CaP-RR cells compared with CaP cells (Figure 1c). In addition, the expression of Beclin-1 and LC3A/B, Ku70 and Ku80 as well as BRCA-1, BRCA-2 and Rad-51 was found to be increased in CaP-RR cells compared with CaP cells (Figure 1c). In the meantime, the *γ*H2AX levels (double-strand break (DSB) marker) were significantly reduced in CaP-RR cells compared with CaP cells (Figure 1c). All quantitative results and *P*-values are summarized in Supplementary Figure S1 and Supplementary Table S2.

### Cytotoxicity of dual or single inhibitors on CaP-RR and CaP cells *in vitro*

Each cell line displayed a variable response to four inhibitors after 24–72 h treatments using MTT assay and no cytotoxic effect was found for vehicle control (chloroform or DMSO) in all cell lines tested. Dose-dependent cell proliferation inhibition by four inhibitors treated for 24 h was observed in each cell line (Supplementary Figure S2). The IC_50_ values at 24 h for CaP-RR and CaP cell lines are summarized in Supplementary Table S3. We found that both CaP-RR and CaP cells are more sensitive to four PI3K and mTOR inhibitors than normal RWPE-1 prostate cells (*P*<0.05), and that CaP-RR cells are less sensitive to four inhibitors than CaP cells in all CaP cell lines (1.5- to 2.5-fold; *P*<0.05; Supplementary Table S3). On the basis of our previously similar studies,^14,15^ we chose the ^1^/_2_IC_50_ values at 24 h for our following experiments in the current study.

### Effect of combination treatment with dual or single PI3K/mTOR inhibitors on colony formation in CaP-RR cells

**Figure 2:**
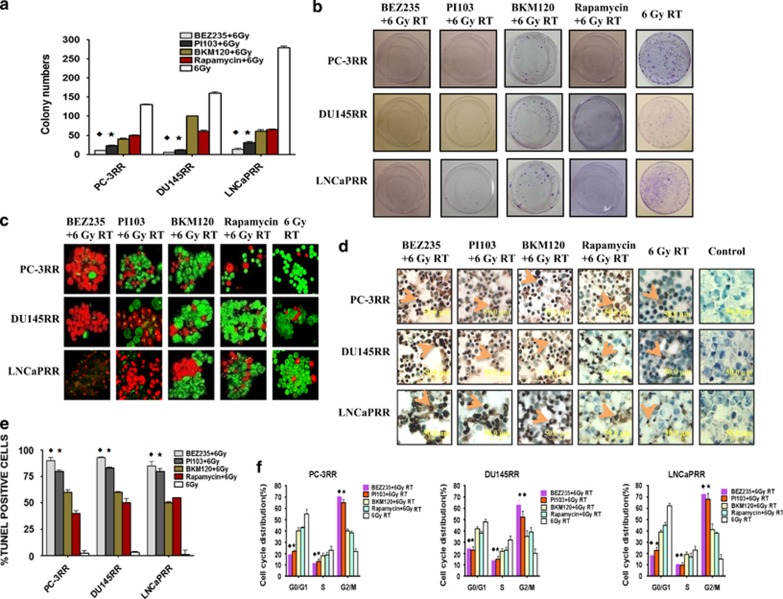
Effects of combination treatment with inhibitors and RT or RT alone on colony formation, apoptosis and cell cycle distribution in CaP-RR cells. (**a**) Colony formation capability was significantly reduced in combination treatment with a dual PI3K/mTOR inhibitor (BEZ235 or PI103) and RT compared with combination of single inhibitor (BKM120 or Rapamycin) and RT or RT alone in CaP-RR cells. (**b**) Typical images of colony growth for the different treatments are shown. Images were taken using a Sony camera (Tokyo, Japan). (**c**) Combination treatment with dual PI3K/mTOR inhibitors and RT induced more apoptotic cells with condensed nuclei (red color) compared with combination with single PI3K/mTOR inhibitors and RT or RT alone in CaP-RR cells while living cells show green. Typical images for AO/EB staining are shown. Magnification × 60 in all images. (**d**) Condensed and fragmented nuclear chromatin characteristics of apoptosis are further confirmed with the TUNEL assay in combination treatment with dual PI3K/mTOR inhibitors and RT in CaP-RR cells. Arrows indicate nuclei (brown). Cells with brown staining are TUNEL-positive cells, whereas blue color indicates normal cancer nuclei. Typical images of TUNEL staining for the different treatments are shown. Magnification × 60 in all images. (**e**) TUNEL-positive cells were significantly increased in combination treatment with dual PI3K/mTOR inhibitors (BEZ235 or PI103) and RT compared with combination of single inhibitor (BKM120 or Rapamycin) and RT or RT alone in CaP-RR cells. (**f**) CaP-RR cells were treated with a dual or single inhibitor for 24 h and then treated with 6 Gy RT or directly treated with 6 Gy RT alone, and cell cycle distributions were analyzed with flow cytometry. Obvious cell cycle arrest in G2/M phase and reduction in G0/G1 and S phases were observed in CaP-RR cells treated with combination with dual inhibitors and RT. In all experiments, ♦ indicates the difference between combination of BEZ235 with RT and combination of single inhibitor (BKM120 or Rapamycin) with RT or RT alone in CaP-RR cells (*P*<0.05). * indicates the difference between combination of PI103 with RT and combination of single inhibitor (BKM120 or Rapamycin) with RT or RT alone in CaP-RR cells (*P*<0.05). All results were from three independent experiments (*n*=3)

Our results indicate that combination treatment with each dual inhibitor (BEZ235 or PI103) and 6 Gy RT consistently showed significant reduction in colony formation, when compared with the CaP-RR cells treated with combination of each single inhibitor (BKM120 or Rapamycin) and 6 Gy RT or 6 Gy RT alone (*P*<0.05), and no significant difference was found between two dual-inhibitor combination treatments (*P*>0.05), although the colony number of combination with BEZ235 and RT was slightly lower than that of PI103 with RT (Figure 2a). No significant difference for colony formation was found between two single-inhibitor combination treatments (Figure 2a) (*P*>0.05). The typical images for colony formation from different treatments are shown in Figure 2b.

We found that the colony growth ability of cells with combination of each inhibitor and RT was significantly lower than that with each inhibitor treatment alone (*P*<0.05). We also compared the plating efficiency of combination of inhibitors and RT to the sum of plating efficiency of inhibitors alone and 6 Gy RT alone and found that the plating efficiency of combination therapy was lower than the sum of the plating efficiency of inhibitors alone and RT alone (*P*<0.05), indicating that the growth-inhibiting effect of the inhibitors is not only because of the direct inhibition but also because of the radiosensitization effect of the inhibitors. The typical images and data for colony formation from different treatments are shown in Supplementary Figure S3.

### Comparison of the effect of combination treatment with dual or single PI3K/mTOR inhibitors on apoptosis in CaP-RR cells

The characteristic morphological changes in the treated CaP-RR cells were found by AO/EB staining, which showed typical features of apoptosis in the combination treatment with dual inhibitors and RT including nuclear condensation and fragmentation compared with combination treatment with single inhibitors and RT, and these changes are not shown in 6 Gy RT alone treated CaP-RR cells (Figure 2c). The apoptosis detected in AO/EB staining was further confirmed with the TUNEL assay (Figure 2d). In TUNEL assay, CaP-RR cells treated by dual inhibitors (BEZ235 or PI103) combined with RT displayed characteristic apoptotic morphology with nuclear chromatin condensation and fragmentation, whereas those treated by single inhibitors (BKM120 or Rapamycin) combined with RT showed less apoptotic cells and almost no apoptotic cells were found in CaP-RR cells exposed to 6 Gy RT alone (Figure 2d). The significant differences for TUNEL-positive cells were observed between combination treatment of dual inhibitors (BEZ235 or PI103) with 6 Gy RT and combination treatment of single inhibitors (BKM120 or Rapamycin) with 6 Gy RT or 6 Gy RT alone in CaP-RR cell lines (*P*<0.05; Figure 2e).

**Figure 3:**
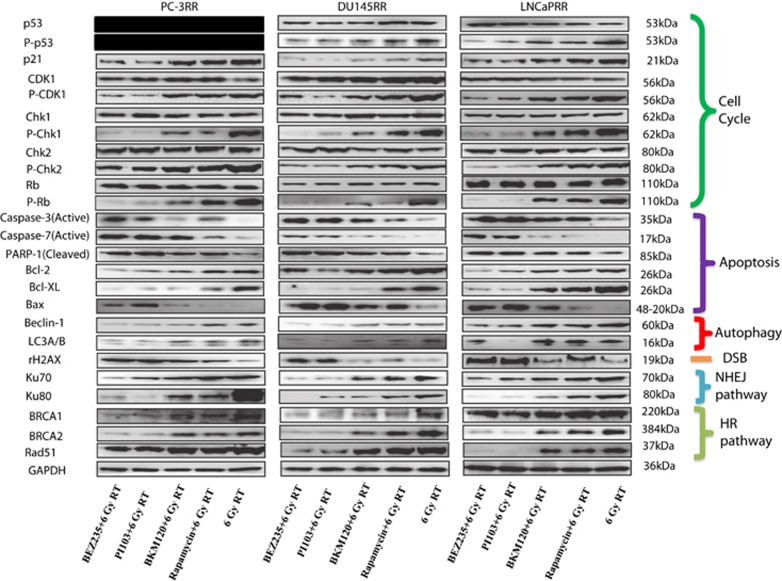
Effects of combination treatment with inhibitors and RT or RT alone on cell cycle check point, apoptosis, autophagy, DSB, NHEJ and HR pathway-related proteins in CaP-RR cells. Dual inhibitors (BEZ235 and PI103) combined with 6 Gy RT effectively induced cell cycle redistribution and high levels of apoptosis and DSB proteins, and reduced autophagy and HR pathway proteins in CaP-RR cells compared with the combination of single inhibitors (BKM120 or Rapamycin) with 6 Gy RT. Cell cycle-related proteins (p-p53, p21, p-CDK1, p-Chk1, p-Chk2 and p-Rb), anti-apoptosis proteins (Bcl-2 and Bcl-xl), autophagy proteins (Beclin-1 and LC3A/B), DSB marker (*γ*H2AX), NHEJ proteins (Ku70 and Ku80) and HR pathway-related proteins (BRCA1, BRCA2 and RAD51) were significantly reduced, whereas no change was found in p53, CDK1, Chk1, Chk2 and Rb proteins. Apoptosis pathway proteins (active caspase-3, active caspase-7, cleaved PARP-1 and Bax) were significantly increased in combination with dual inhibitors and RT compared with combination with single inhibitors and RT or RT alone. All protein expression levels were determined with western blot analysis. GAPDH was used as a loading control. Typical images are shown from three independent experiments (*n*=3)

Compared with combination treatment with single inhibitors and RT, combination treatment with dual inhibitors and RT can induce high levels of active capase-3, active caspase-7 and cleaved PARP-1 in three CaP-RR cell lines, although all combination treatments including dual inhibitors and single inhibitors with RT increased the expression of the active capase-3, active caspase-7 and cleaved PARP-1 compared with RT alone (Figure 3). We also found the increased trend for Bcl-2 and Bcl-xl, and reduced trend for Bax from combination treatment with dual inhibitors and RT, combination treatment with single inhibitors and RT to 6 Gy RT alone in all three CaP-RR cell lines (Figure 3).

### Combination treatment affects cell cycle distribution and inactivates cell cycle check point proteins in CaP-RR cells

We found that the significant reduction in G0/G1 and S phases and obvious increase in G2/M phase were found in combination treatment with dual inhibitors and RT compared with combination treatment with single inhibitors and RT or RT alone in all three CaP-RR cells (*P*<0.05), that statistical difference was also found between combination treatment with single inhibitors and RT and RT alone for cell cycle distribution in all three CaP-RR cells (*P*<0.05), and that no difference was seen between two dual inhibitors (or two single inhibitors) with RT (*P*>0.05; Figure 2f). The details of cell cycle redistributions after combination treatments are summarized in Supplementary Table S4.

To investigate the role of check point proteins in cell cycle redistribution in the combination treatment, we found that the expression of Ki67 became very weak after combination treatment with dual inhibitors and RT, especially in PC-3RR and LNCaPRR cells compared with other treatments, and no significant change was found between single inhibitors combined with RT and RT alone in three CaP-RR cell lines (data not shown). We also demonstrated that the expression of p-p53 and p21 was obviously reduced, whereas no significant change was seen in the expression of p53 in DU145RR and LNCaPRR cell lines after treatment by dual inhibitors with RT, as p53 is negative in PC-3 cells (Figure 3).

The expression levels of CDK1, Chk1, Chk2 and Rb did not obviously alter in each treatment; however, combination treatment with dual inhibitors and RT led to markedly diminished levels of p-CDK1, p-Chk1, p-Chk2 and p-Rb compared with other treatments in CaP-RR cells (Figure 3). The findings from cell check point proteins are consistent with those from cell cycle redistribution after combination treatment. All quantitative results and *P*-values are summarized in Supplementary Figure S4 and Supplementary Tables S5.

### Effect of combination treatment with dual or single PI3K/mTOR inhibitors on autophagy and DNA repair pathways in CaP-RR cells

The expression of Beclin-1 and LC3A/B proteins (autophagy markers) was found to be significantly reduced, whereas the *γ*H2AX level (DSB marker) was found to be significantly increased in combination treatment with dual inhibitors and RT compared with combination treatment with single inhibitors and RT or RT alone in three CaP-RR cell lines (Figure 3). Accordingly, the expression of NHEJ (Ku70 and Ku80) and HR (BRCA-1, BRCA-2 and Rad-51) repair pathway proteins in combination treatment with dual inhibitors and RT was markedly reduced in CaP-RR cells (Figure 3). All quantitative results and *P*-values are summarized in Supplementary Figure S4 and Supplementary Tables S5.

## Discussion

**Figure 4:**
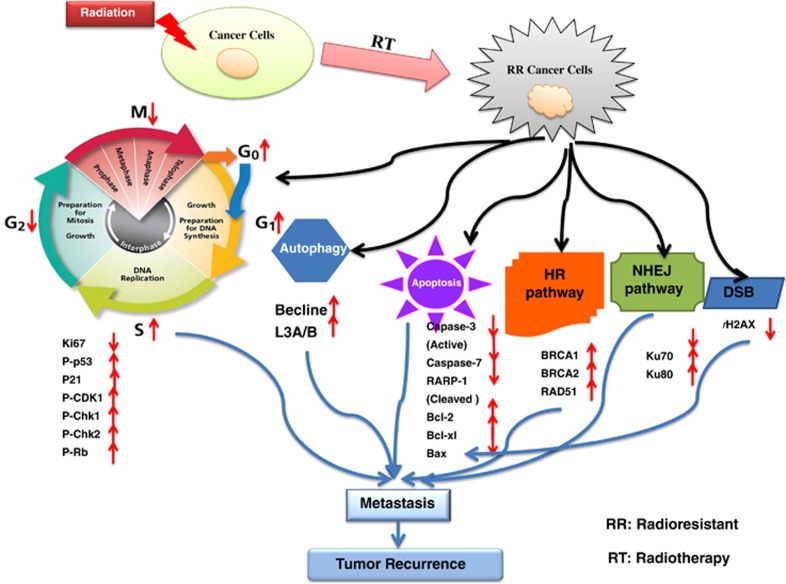
Diagram showing that CaP-RR cells are associated with the induction of cell cycle redistribution, inactivation of apoptosis proteins, activation of cell cycle checkpoint, autophagy, DSB, NHEJ and HR DNA repair pathway proteins compared with CaP cells (control)

**Figure 5:**
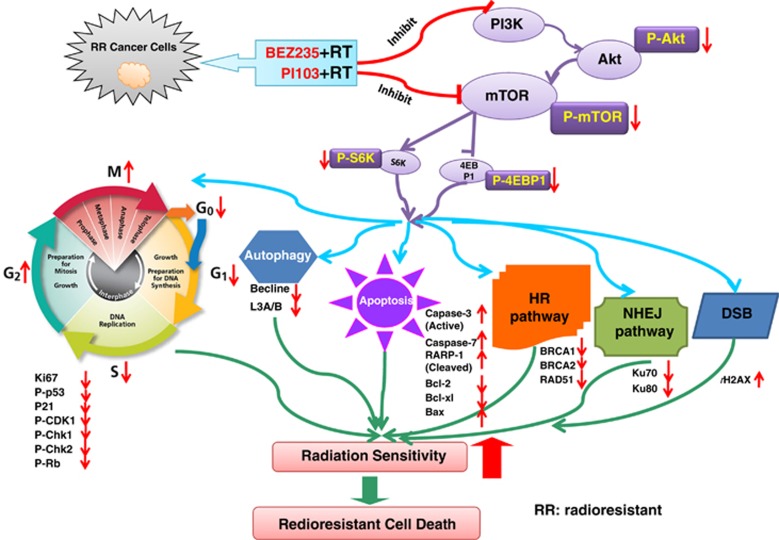
Diagram showing that the model proposed for two dual PI3K/mTOR inhibitors (BEZ235 or PI103) combined with RT induces cell cycle redistribution and apoptosis, increases DNA DSB, reduces autophagy, inactivates NHEJ and HR repair pathways, and enhances radiosensitivity in CaP-RR cells. The results from combination treatment with dual inhibitors and RT were compared with that with single inhibitors and RT or RT alone

In the current study, using CaP-RR model and cancer cell biology techniques, we present novel insight into the effects of a combination treatment with PI3K/mTOR inhibitors and RT as well as the putative mechanisms. In the first step, we demonstrate the association of CaP-RR cells with cell cycle distribution, cell cycle check point proteins, apoptosis and autophagy proteins, and DNA repair pathway proteins as shown in Figure 4. Our findings from the treatment of CaP-RR cells with a combination of two dual PI3K/mTOR inhibitors (BEZ235 and PI103) and RT are summarized in the model presented in Figure 5.

Cells in different phases of the cell cycle exhibit differential radiation sensitivity. In general, cells are most sensitive to radiation-induced DNA damage during G2/M, and cells in late S phase are the most resistant ones to ionizing radiation.^16^ According to the report by Hoppe *et al*,^17^ cells are most radiosensitive in G2 and M phases but most RR in the S phase, whereas for cells with the long cycle, it also shows radioresistance in early G1. Tell *et al.*^18^ reported that S-phase cells are highly increased in lymphocytes of patients showing no response to RT compared with lymphocytes of partial and complete responders. Our current data in cell cycle analysis are consistent with these previous reports and are also in line with our previous proliferation study and enhanced CSC phenotypes in these CaP-RR cells,^7^ as CSCs are quiescent cells with a very lower proliferation rate.^19^

Ki67 is a nuclear protein associated with cellular proliferation. We found that Ki67 expression was much lower in CaP-RR cells than in CaP cells, which may partially explain the G0/G1 arrest in this study. In our previous study, we demonstrated that the p53-p21 axis has a predominant role in the regulation of cell cycle in CaP RT.^15^ P21 protein regulates each cyclin-CDK (such as CDK1) complex at G1 and S phases,^20^ inhibiting CDK1 phosphorylation, thereby leading to a G2/M cell cycle arrest.^21^ Chk1/2 activation mediated by p53 phosphorylation leads to G1 arrest.^22^ Our current study indicated that the increase in p-p53, p21 and p-Chk1/2 in CaP-RR cells may be in accordance with cell cycle G1 arrest, whereas the enhancement of p-CDK1 in CaP-RR cells could be associated with the reduction of G2/M phases. However, it was reported that p21 activation inhibits p-CDK1 and results in G2/M arrest,^21^ suggesting that p-CDK1 may be regulated by alternative pathway mechanisms and different cancer types may be regulated by different pathways. In addition, p-Rb, as an important cell cycle checkpoint protein, was also increased (activated) in CaP-RR cells, indicating that this protein is also involved in CaP radioresistance. Rb protein is essential in the G1 phase of the cell cycle and is a crucial checkpoint responsible for G2/M arrest of cancer cells to radioresistance.^23^ The activation of Rb can also explain the G0/G1 arrest and G2/M reduction. All the findings suggest that a panel of cell cycle check point proteins are responsible for CaP radioresistance.

**Figure 6:**
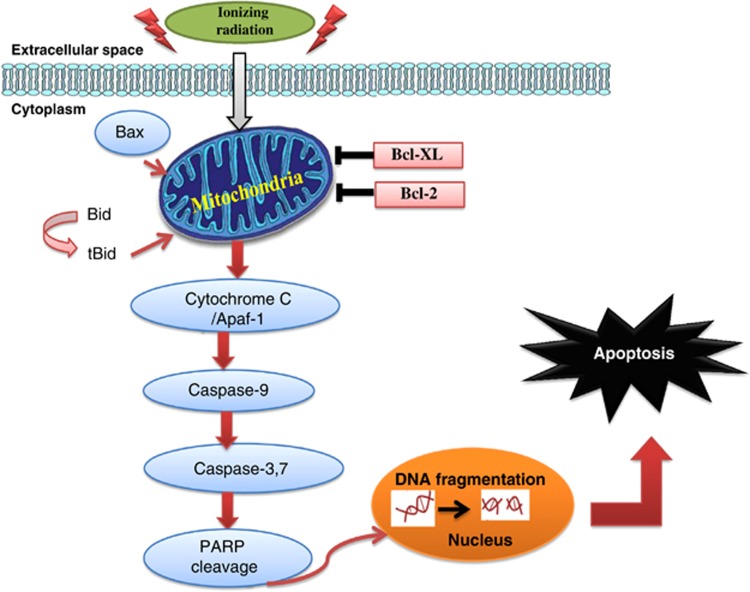
Diagram showing that radioresistance is associated with repression of apoptosis pathway by upregulation of Bcl-XL and Bcl-2 and downregulation of Caspase-3, Caspase-7, Cleaved PARP and Bax in CaP-RR cells

**Figure 7:**
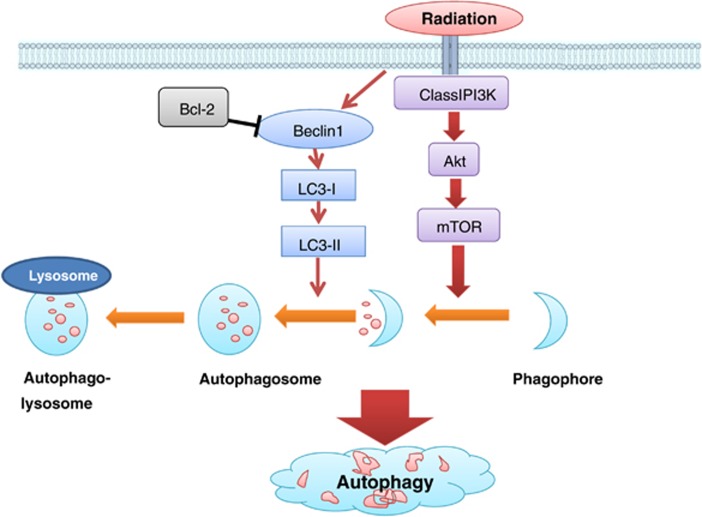
Diagram showing that radioresistance is associated with induction of the autophagy pathway by activation of Beclin and LC3A/B in CaP-RR cells

Apoptosis has a crucial role in cell death after RT, and autophagy is called as ‘the second apoptosis'. In cancer therapy, the role of autophagy is paradoxical, in which this cellular process may serve as a pro-survival or pro-death mechanism to counteract or mediate the cytotoxic effect of anticancer agents.^24^ Our current data support that CaP radioresistance is associated with apoptosis and autophagy pathways and that autophagy promotes CaP-RR cell survival. The schematic diagrams of the correlations between RT and apoptosis or autophagy in CaP-RR cells are shown in Figures 6 and 7, respectively.

The DNA DSB is the principle cytotoxic lesion for ionizing radiation. Two main pathways are responsible for DNA DSB repair, which are NHEJ and HR.^25^ We found that the key proteins including Ku70 and Ku80 (NHEJ pathway) as well as BRCA1, BRCA2 and Rad51 (HR pathway) are activated, whereas *γ*H2AX was reduced in CaP-RR cells, which implies that the NHEJ and HR repair pathways have an important role in the regulation of CaP radioresistance after exposure to RT.

The PI3K/Akt/mTOR signaling pathway is important for cancer metastasis and radioresistance. Using a label-free quantitative liquid chromatography/tandem mass spectrometry (LC-MS/MS) proteomic approach, we have identified the PI3K/Akt/mTOR signaling pathway as the main pathway associated with radioresistance in three CaP-RR cell lines (PC-3RR, DU145RR and LNCaPRR) developed in our laboratory (unpublished data), further confirming the importance of this pathway in CaP radioresistance. In the current study, we chose two dual PI3K/mTOR inhibitors (BEZ235 and PI103) and two single inhibitors (BKM120 and Rapamycin) as radiosensitizers to compare their effects in the treatment of CaP-RR cells. The reasons are as follows: (1) it was reported that the use of dual inhibitors of PI3K and mTOR is a promising approach and can more efficiently inhibit the PI3K/Akt/mTOR pathway than single PI3K or mTOR inhibitor and produce better treatment outcome;^26^ (2) as three out of four inhibitors selected in this study have been used in clinical trials, if successful, the combination of these inhibitors and RT can be easily developed in *in vivo* animal study and clinical trials; (3) we were interested to know whether a combination of a dual inhibitor with RT is more effective than a combination of a single inhibitor with RT for the treatment of CaP-RR cells.

In the current study, we found that both CaP-RR and CaP cells are more sensitive to four inhibitors than the normal prostate RWPE-1 cells, and that CaP cells are more sensitive than CaP-RR cells (Supplementary Table S1), suggesting that PI3K/mTOR inhibitors more selectively target cancer cells but not normal cells and that CaP-RR cells are more resistant to these inhibitors. In the next step, we found that combination with dual inhibitors (BEZ235 and PI103) and 6 Gy RT can greatly repress tumor colony growth, induce more apoptosis and improve radiosensitivity compared with combination with dual inhibitors (BMK120 and Rapamycin) and 6 Gy RT (*P*<0.05). The finding from the reduced colony growth in the combination of dual inhibitors and RT is consistent with the significant reduction of Ki67 expression and the activation of more apoptosis pathway proteins (see Figure 3). One possible reason for dual PI3K/mTOR inhibitors inducing more radiosensitivity could be that dual PI3K/mTOR inhibitors have a broader efficacy across more genotypes with pro-apoptotic effects identified in a wider range of cell lineages compared with agents targeting only one component of the pathway.^27,28^ Another possible reason could be that dual inhibitors of PI3K and mTOR target the active sites of both holoenzymes, inhibiting the pathway both upstream and downstream of Akt, thus avoiding the problem of Akt activation following abolition of the mTORC1-S6K-IRS1-negative feedback loop, which is known to occur with single mTOR inhibitors.^27^

Combined together with the reduced colony growth and increased apoptosis, we also found that combination of dual inhibitors (BEZ235 and PI103) with RT greatly changed cell cycle distribution and caused higher cell arrest in G2/M phase (the most sensitive phase to radiation) and reduction of cells in G0/G1 and S phases (the most resistant phases to radiation) compared with combination of single inhibitors (BKM120 and Rapamycin) with RT. The results in cell cycle redistribution were further confirmed by cell cycle checkpoint protein alteration. We found that the levels of p-p53, p21, p-CDK1, p-Chk1, p-Chk2 and p-Rb proteins were much lower in combination of BEZ235 or PI103 and RT compared with combination of BKM120 or Rapamycin with RT or RT alone, which is consistent with G2/M arrest in cell cycle arrest. As the p53–p21 axis is important in the regulation of CaP radiosensitivity, the deficiency of p53 in PC-3 cells suggests that p21 may be regulated by alternative mechanisms in this cell line.^15^ These results indicate that combination treatment with dual inhibitor and RT can obviously change cell cycle distribution in G0/G1, S and G2/M phases and greatly affect cell cycle check point proteins, which is consistent with the observations in colony growth and apoptosis.

The role of autophagy in RT remains controversial. Chang *et al.*^29^ found that induction of autophagy by BEZ235 may be a survival mechanism that counteracts its anticancer effects. Using RR pancreatic cancer cell lines, Wang *et al.*^30^ showed that reduced levels of the miR-23b increase levels of ATG12 and autophagy to promote radioresistance. In this study, obviously reduced expression of Beclin-1 and LC3A/B in combination of BEZ235 or PI103 with RT compared with single inhibitor combined with RT or RT alone further confirms that autophagy is involved in CaP radioresistance and that reduced autophagy proteins are associated with increased radiosensitivity in CaP-RR cells after combination treatment.

In our study, *γ*H2AX was used as a biomarker to measure DNA DSB because *γ*H2AX is a highly specific and sensitive molecular marker for monitoring both DSB initiation and resolution.^31^ We found that phosphorylation of histone *γ*H2AX was enhanced by combination treatment with dual or single inhibitors and RT and that combination of dual inhibitors with RT induced more DNA breaks and concomitantly greatly reduced the NHEJ and HR repair pathway proteins compared with a single inhibitor combined with RT or RT alone, suggesting that both NHEJ and HR repair pathways are the main DNA repair pathways involved in radiosensitization effect induced by dual or single inhibitors and RT in CaP-RR cells.

CSCs are becoming recognized as being responsible for metastasis and radioresistance, and are thought to be in a relatively quiescent state, thus evading radiotherapeutic challenges and ‘protecting' the continuity of the tumor. The CSC model has clinical implications, in that CSCs have been known to contribute to radioresistance predominantly through enhanced levels of DNA repair activity and slow cell cycle kinetics. In this study, our CaP-RR cells with induced EMT and enriched CSCs^7^ could be successfully treated with combination of dual PI3K/mTOR inhibitors and RT, suggesting that this combination therapy may target CSCs (‘root' of cancer recurrence) to overcome radioresistance and prevent metastasis. Therefore, our current findings may have clinical significance in CaP RT. Studying the effects of the combination of dual PI3K/mTOR inhibitor with RT in CaP-RR xenograft animal models is ongoing in our laboratory now.

In conclusion, our study demonstrates for the first time that CaP-RR cells are associated with cell cycle arrest in G0/G1 and S phases, inactivation of apoptosis pathway proteins, activation of cell cycle checkpoint, autophagy, NHEJ and HR repair pathway proteins; that combination of the dual PI3K/mTOR inhibitors with RT can greatly repress tumor colony growth, induce more apoptosis and improve radiosensitivity. The putative mechanisms of the radiosensitization effect in CaP-RR cells in the combination treatment include cell cycle redistribution to a more radiosensitive phase (G2/M) and abolishment of RR cell cycle arrest phase (G0/G1 and S), activation of apoptosis death pathway, inhibition of autophagy survival pathway, induction of more DNA damage and inhibition of repair of RT-induced DNA DSBs through diminishing NHEJ and HR pathways. Our findings suggest that combination with a dual PI3K/mTOR inhibitor (BEZ235 or PI103) is a promising treatment modality for future CaP RT.

## Materials and Methods

### Antibodies and reagents

Antibodies were obtained from different sources. The detailed information and conditions for all antibodies are listed in Supplementary Table S8. The details for 4 PI3K/mTOR inhibitors are summarized in Supplementary Table S9.

### Cell line and cell culture

Human CaP cell lines (PC-3, DU145 and LNCaP) and human prostate epithelial cell line (RWPE-1) were obtained from American Type Culture Collection (Rockville, MD, USA). Three CaP-RR sublines including PC-3RR, DU145RR and LNCaPRR were developed in our laboratory using a low dose of radiation treatment.^7^ The identity for all cell lines was confirmed using a short tandem repeat profiling by CellBank, Sydney, NSW, Australia in 2013. All cell culture reagents were supplied by Invitrogen Australia Pty Ltd, Melbourne, VIC, Australia unless otherwise stated. The cell lines were cultured as previously described conditions.^7^

### *In vitro* cell cytotoxicity assay

Cell cytotoxicity was evaluated in CaP-RR (PC-3RR, DU145RR and LNCaPRR) and CaP (PC-3, DU145 and LNCaP) cell lines after treatment with inhibitors (BEZ235, PI103, BKM120 and Rapamycin) using MTT assay following the published method.^7^ The IC_50_ (50% inhibitory concentrations) of each inhibitor in CaP-RR cell lines were calculated.

### Flow cytometric analysis for cell cycle distribution

A flow cytometry assay was performed using a published method.^15^ This assay was performed for comparison of cell cycle distribution between CaP-RR and CaP cells or for comparison of the difference after different treatments in CaP-RR cells.

(1) For comparison of cell cycle between CaP-RR and CaP cells, briefly, cells (1 × 10^6^) were seeded in a 75-cm^2^ flask for 48 h. Trypsinized adherent and floating cells were pooled and fixed in a cold 70% (v/v) ethanol at 4 °C overnight (o/n) and then resuspended in PBS before staining with FxCycleViolet (Life technologies, Melbourne VIC, Australia) for 30 min at room temperature. Each sample contained 1-ml cell suspension with 1 × 10^6^ cells and 1-μl FxCycleViolet stain. Analysis was performed at 405 nm excitation and emission collected with a 450/50 band-pass filter using a FACSCanto ll Flow Cytometer (Becton, Dickinson and Company, BD Biosciences, San Jose, CA, USA). Histograms of DNA content were analyzed using the FlowJo software (V.7.6.1, Tree Star Inc., Ashland, OR, USA) to determine cell cycle distribution (subs G0, G1, S and G2/M).

(2) For comparison of cell cycle difference after different treatments, CaP-RR cells (1 × 10^6^) were cultured for 48 h as above, treated with dual or single PI3K/mTOR inhibitors at the respective ^1^/_2_IC_50_ concentrations for 24 h and then treated with 6 Gy RT, or treated with 6 Gy RT alone as a control. The treated cells were prepared as above for analysis.

### Clonogenic survival assay

CaP-RR cells were used for colony-forming assays following the published method.^7^

### Detection of apoptosis

CaP-RR cells (5 × 10^5^) were cultured in 25-cm^2^ flasks for 24 h and then treated with combination treatment with ^1^/_2_IC_50_ inhibitor and RT (6 Gy) or single RT (6 Gy) or vehicle control (chloroform and DMSO) as described in the Flow cytometry analysis section. The treated cells were performed for AO/EB staining^14^ and TUNEL assay^32^ to evaluate apoptosis.

### Western blotting analysis

Cultured CaP-RR and CaP cells or CaP-RR cells with different treatments as mentioned above in flow cytometry analysis were prepared. Protein expression levels were determined with western blot analysis as previously described.^7^ Different primary antibodies used are shown in Supplementary Table S8. To confirm equal loading of protein lysates, membranes were stripped (Restore Western Blot Stripping Buffer, Thermo Fisher Pierce, Scoresby, VIC, Australia) and re-probed using a mouse anti-*β*-tubulin monoclonal antibody or mouse anti-GAPDH polyclonal antibody.

### Statistical analysis

All experiments were performed at least three times (*n*=3). All numerical data were expressed as the average of the values (mean), and the S.D. was calculated. Possible significant differences (*P*<0.05) were evaluated using the two-tailed Student's *t*-test with the GraphPad Prism 4.00 (GraphPad, San Diego, CA, USA).

## Abbreviations

CSC: cancer stem cell
DSB: double-strand break
EMT: epithelial–mesenchymal transition
HR: homologous recombination
NHEJ: non-homologous end joining
CaP: prostate cancer
RT: radiotherapy
RR: radioresistant
