# Supplementary material for: PI3K/Akt/mTOR pathway inhibitors enhance radiosensitivity in radioresistant prostate cancer cells through inducing apoptosis, reducing autophagy, suppressing NHEJ and HR repair pathways
Source: Cell Death Dis. 2014 Oct 2;5(10):e1437–. doi: 10.1038/cddis.2014.415 (PMC4237243; doi:10.1038/cddis.2014.415)
Supplement: Supplementary Table S1 [file cddis2014415x1.doc]

**Table S1. Difference of cell cycle distribution between CaP-RR and CaP cell lines**

| **Cell line** | **Condition** | **Phase** | | | | | |
| --- | --- | --- | --- | --- | --- | --- | --- |
|  |  | G0/G1 | | S | | G2/M | |
|  |  | Mean (%) | SD | Mean (%) | SD | Mean (%) | SD |
| PC-3 | RR | 60.1^🟉^ | 5.1 | 29.2^🟉^ | 4.0 | 10.7^🟉^ | 2.3 |
|  | control | 48.1 | 3.2 | 20.4 | 2.0 | 31.5 | 0.3 |
| DU145 | RR | 62.5^🟉^ | 6.8 | 31.1^🟉^ | 3.1 | 6.4^🟉^ | 4.1 |
|  | control | 51.2 | 5.1 | 19.2 | 1.6 | 29.6 | 3.2 |
| LNCaP | RR | 63.8^🟉^ | 3.5 | 27.1^🟉^ | 4.1 | 9.1^🟉^ | 5.2 |
|  | control | 52.3 | 2.2 | 17.2 | 2.1 | 30.5 | 1.9 |

**Notes**: ^🟉^ indicates that a significant difference is found between CaP-RR and CaP cell lines in G0/G1, S and G2/M phases using flow cytometry analysis (*P*<0.05).
