# Supplementary material for: PI3K/Akt/mTOR pathway inhibitors enhance radiosensitivity in radioresistant prostate cancer cells through inducing apoptosis, reducing autophagy, suppressing NHEJ and HR repair pathways
Source: Cell Death Dis. 2014 Oct 2;5(10):e1437–. doi: 10.1038/cddis.2014.415 (PMC4237243; doi:10.1038/cddis.2014.415)
Supplement: Supplementary Table S2 [file cddis2014415x2.doc]

|  | ***P* value** | | |
| --- | --- | --- | --- |
|  | **PC-3 VS PC-3RR** | **DU145 VS DU145RR** | **LNCaP VS LNCaPRR** |
| p53 | N/A | 0.95 | 0.99 |
| P-p53 | N/A | 0.49×10^-2^ | 0.77×10^-1^ |
| p21 | 2.72×10^-6^ | 1.63×10^-5^ | 0.23×10^-1^ |
| CDK1 | 0.14 | 0.75 | 0.97 |
| P-CDK1 | 1.59×10^-5^ | 0.1×10^-3^ | 0.26×10^-3^ |
| Chk1 | 0.29 | 0.38 | 0.49 |
| P-Chk1 | 2.35×10^-5^ | 0.16×10^-2^ | 0.14×10^-3^ |
| Chk2 | 0.59 | 0.36 | 0.53 |
| P-Chk2 | 1.15×10^-8^ | 4.99×10^-7^ | 0.51×10^-3^ |
| Rb | 0.79 | 0.33 | 0.49 |
| P-Rb | 1.07×10^-8^ | 1.51×10^-11^ | 4.56×10^-8^ |
| active caspase-3 | 8.12×10^-5^ | 0.18×10^-3^ | 3.64×10^-5^ |
| active caspase-7 | 6.08×10^-5^ | 1.54×10^-5^ | 5.93×10^-5^ |
| cleaved PARP-1 | 0.18×10^-3^ | 0.19×10^-3^ | 0.69×10^-3^ |
| Bcl-2 | 0.44×10^-2^ | 5.53×10^-5^ | 0.43×10^-2^ |
| Bcl-xl | 0.12×10^-2^ | 0.13×10^-3^ | 0.2×10^-2^ |
| Bax | 0.11×10^-2^ | 0.17×10^-2^ | 0.88×10^-3^ |
| Beclin-1 | 0.19×10^-3^ | 7.78×10^-5^ | 8.11×10^-5^ |
| LC3A/B | 0.11×10^-2^ | 0.55×10^-3^ | 0.86×10^-3^ |
| γH2AX | 7.31×10^-5^ | 0.42×10^-1^ | 0.45×10^-1^ |
| Ku70 | 0.22×10^-2^ | 3.62×10^-5^ | 1.9×10^-5^ |
| Ku80 | 0.19×10^-3^ | 0.23×10^-3^ | 3.56×10^-5^ |
| BRCA1 | 2.1×10^-5^ | 0.26×10^-3^ | 0.12×10^-1^ |
| BRCA2 | 0.14×10^-3^ | 0.19×10^-3^ | 0.46×10^-3^ |
| RAD51 | 0.12×10^-2^ | 3.97×10^-5^ | 5.48×10^-5^ |

**Table S2. Summary of P values for protein fold variation of CaP-RR cells in relative to CaP-control cells**

|  |
| --- |

**Note**: N/A means “not applicable”.
