# Supplementary material for: PI3K/Akt/mTOR pathway inhibitors enhance radiosensitivity in radioresistant prostate cancer cells through inducing apoptosis, reducing autophagy, suppressing NHEJ and HR repair pathways
Source: Cell Death Dis. 2014 Oct 2;5(10):e1437–. doi: 10.1038/cddis.2014.415 (PMC4237243; doi:10.1038/cddis.2014.415)
Supplement: Supplementary Table S3 [file cddis2014415x3.doc]

**Table S3.** IC_50_values for dual inhibitors (BEZ235 and PI103) and single inhibitor (BKM120 and Rapamycin)

tested by MTT assay in CaP-RR and CaP cell lines for 24 hours treatment

| **Cell line** | **^⬥^PC-3RR** | **PC-3** | **^⬥^DU145RR** | **DU145** | **^⬥^LNCaPRR** | **LNCaP** | **^🟉^RWPE-1** |
| --- | --- | --- | --- | --- | --- | --- | --- |
| **BEZ235(nM)** | 160.2±3.1 | 80.1±2.3 | 135.5±5.6 | 70.3±1.2 | 100.8±7.2 | 80.6±0.3 | 507.5±3.5 |
| **PI103(nM)** | 273.1±2.8 | 195.2±0.6 | 521.2±3.1 | 230.4±0.3 | 337.4±4.9 | 210.7±1.9 | 742.0±2.4 |
| **BKM120(µM)** | 48.8±0.5 | 36.2±0.3 | 107.3±0.5 | 87.3±0.8 | 90.4±2.1 | 60.3±0.12 | 112.3±7.0 |
| **Rapamycin(nM)** | 61.3±3.5 | 39.0±1.2 | 30.1±0.4 | 12.0±0.01 | 20.1±0.4 | 15.8±0.06 | 95.6±1.6 |

**Notes:** IC_50_ value indicates when a range of inhibitor concentrations were used, the concentration of inhibitor was

calculated for 50% cell killing. The results (mean of IC_50_) are from three independent experiments (n=3).

**⬥** indicates that a significant difference is found between CaP-RR and CaP cell lines using 4 different inhibitors (*P*<0.05).

**🟉** indicates that a significant difference is found between prostate normal cell RWPE-1 and prostate cancer cells using 4

different inhibitors (*P*<0.05).
