# Supplementary material for: PI3K/Akt/mTOR pathway inhibitors enhance radiosensitivity in radioresistant prostate cancer cells through inducing apoptosis, reducing autophagy, suppressing NHEJ and HR repair pathways
Source: Cell Death Dis. 2014 Oct 2;5(10):e1437–. doi: 10.1038/cddis.2014.415 (PMC4237243; doi:10.1038/cddis.2014.415)
Supplement: Supplementary Table S4 [file cddis2014415x4.doc]

**Table S4. Cell cycle redistribution after combination with dual or single PI3K/mTOR inhibitors with RT or RT alone in CaP-RR cells**

| **Cell line** | **Treatment** | **Phase** | | | | | |
| --- | --- | --- | --- | --- | --- | --- | --- |
|  |  | G0/G1 | | S | | G2/M | |
|  |  | Mean (%) | SD | Mean (%) | SD | Mean (%) | SD |
| PC-3RR | BEZ235+6 Gy RT | 18.8^⬥^ | 3.1 | 11.2^⬥^ | 2.2 | 70.0^⬥^ | 0.4 |
|  | PI103+6 Gy RT | 21.7^⬥^ | 5.6 | 13.0^⬥^ | 4.0 | 65.3^⬥^ | 2.1 |
|  | BKM120+6 Gy RT | 41.5 | 3.0 | 18.0 | 4.6 | 38.5 | 5.0 |
|  | Rapamycin+6 Gy RT | 43.0 | 3.3 | 18.8 | 4.6 | 38.2 | 1.6 |
|  | 6 Gy RT | 53.1 | 4.6 | 23.9 | 6.7 | 23 | 7.8 |
| DU145RR | BEZ235+6 Gy RT | 24.0^⬥^ | 3.2 | 13.4^⬥^ | 2.1 | 62.6^⬥^ | 8.0 |
|  | PI103+6 Gy RT | 22.9^⬥^ | 5.0 | 14.9^⬥^ | 4.0 | 52.2^⬥^ | 9.0 |
|  | BKM120+6 Gy RT | 40.5 | 3.6 | 22.6 | 4.2 | 35.9 | 9.2 |
|  | Rapamycin+6 Gy RT | 38.0 | 3.9 | 23.1 | 6.3 | 38.9 | 6.9 |
|  | 6 Gy RT | 45.3 | 4.4 | 31.9 | 7.4 | 22.8 | 4.9 |
| LNCaPRR | BEZ235+6 Gy RT | 17.9^⬥^ | 2.1 | 10.1^⬥^ | 0.7 | 72.0^⬥^ | 2.6 |
|  | PI103+6 Gy RT | 22.6^⬥^ | 9.1 | 9.5^⬥^ | 7.3 | 67.9^⬥^ | 2.7 |
|  | BKM120+6 Gy RT | 39.1 | 4.2 | 18.8 | 3.4 | 41.1 | 6.6 |
|  | Rapamycin+6 Gy RT | 45.1 | 2.3 | 17.0 | 7.6 | 37.9 | 5.0 |
|  | 6 Gy RT | 62.1 | 9.5 | 22.9 | 6.1 | 15.0 | 3.5 |

**Notes:** ^⬥^ indicates that a significant difference is found in G0/G1, S and G2/M phases in CaP cells with different treatments using flow cytometry analysis (*P*<0.05).
