# Supplementary material for: PI3K/Akt/mTOR pathway inhibitors enhance radiosensitivity in radioresistant prostate cancer cells through inducing apoptosis, reducing autophagy, suppressing NHEJ and HR repair pathways
Source: Cell Death Dis. 2014 Oct 2;5(10):e1437–. doi: 10.1038/cddis.2014.415 (PMC4237243; doi:10.1038/cddis.2014.415)
Supplement: Supplementary Table S5 [file cddis2014415x5.doc]

|  | **P value** | | | | | |
| --- | --- | --- | --- | --- | --- | --- |
| **PC-3**  **RR cell** | **BEZ235+RT VS BKM120+RT** | **BEZ235+RT VS Rapamycin+RT** | **BEZ235+RT VS**  **6 Gy RT** | **PI103+RT VS BKM120+RT** | **PI103+RT**  **VS Rapamycin+RT** | **PI103+RT VS**  **6 Gy RT** |
| p53 | N/A | N/A | N/A | N/A | N/A | N/A |
| P-p53 | N/A | N/A | N/A | N/A | N/A | N/A |
| p21 | 1.02×10^-5^ | 3.11×10^-6^ | 4.17×10^-5^ | 5.82×10^-5^ | 2.34×10^-5^ | 5.68×10^-5^ |
| CDK1 | 0.9 | 0.91 | 0.49×10^-2^ | 0.28×10^-2^ | 0.18×10^-3^ | 1.18×10^-5^ |
| P-CDK1 | 3.89×10^-6^ | 1.42×10^-6^ | 4.15×10^-8^ | 6.22×10^-6^ | 2.83×10^-6^ | 2.22×10^-7^ |
| Chk1 | 0.28×10^-2^ | 0.45×10^-3^ | 0.3×10^-3^ | 0.15×10^-3^ | 1.72×10^-5^ | 4.80×10^-6^ |
| P-Chk1 | 7.13×10^-6^ | 1.40×10^-5^ | 5.85×10^-7^ | 5.59×10^-7^ | 1.92×10^-6^ | 8.45×10^-8^ |
| Chk2 | 0.17×10^-2^ | 0.68×10^-3^ | 0.41×10^-1^ | 0.65×10^-3^ | 0.29×10^-3^ | 0.60×10^-2^ |
| P-Chk2 | 3.02×10^-5^ | 1.08×10^-5^ | 1.14×10^-7^ | 3.40×10^-5^ | 1.95×10^-5^ | 5.53×10^-7^ |
| Rb | 0.31×10^-1^ | 0.15 | 0.53 | 0.64×10^-2^ | 0.24×10^-2^ | 0.26×10^-2^ |
| P-Rb | 1.96×10^-5^ | 2.77×10^-6^ | 5.18×10^-7^ | 1.74×10^-5^ | 2.21×10^-6^ | 4.44×10^-7^ |
| active caspase-3 | 3.20×10^-7^ | 5.88×10^-7^ | 6.08×10^-7^ | 0.13×10^-1^ | 0.32×10^-1^ | 0.15×10^-2^ |
| active caspase-7 | 2.46×10^-6^ | 1.9×10^-6^ | 1.57×10^-6^ | 1.25×10^-6^ | 1.11×10^-6^ | 1.36×10^-6^ |
| cleaved PARP-1 | 0.74×10^-2^ | 0.14×10^-2^ | 0.13×10^-3^ | 0.55×10^-3^ | 6.37×10^-5^ | 1.88×10^-5^ |
| Bcl-2 | 0.28×10^-2^ | 5.49×10^-5^ | 6.66×10^-5^ | 0.97×10^-2^ | 0.79×10^-3^ | 0.18×10^-3^ |
| Bcl-xl | 0.26×10^-1^ | 2.46×10^-5^ | 1.02×10^-6^ | 0.1×10^-1^ | 1.29×10^-5^ | 4.31×10^-7^ |
| Bax | 0.10×10^-3^ | 4.76×10^-7^ | 3.40×10^-7^ | 1.95×10^-5^ | 1.23×10^-7^ | 1.03×10^-7^ |
| Beclin-1 | 0.59×10^-2^ | 2.12×10^-5^ | 3.13×10^-5^ | 0.14×10^-1^ | 1.25×10^-5^ | 2.49×10^-5^ |
| LC3A/B | 0.95×10^-2^ | 0.17×10^-2^ | 0.52×10^-3^ | 0.45×10^-2^ | 0.27×10^-3^ | 6.54×10^-5^ |
| γH2AX | 6.79×10^-6^ | 3.67×10^-6^ | 1.65×10^-6^ | 5.40×10^-6^ | 2.68×10^-6^ | 1.28×10^-6^ |
| Ku70 | 3.26×10^-5^ | 4.60×10^-6^ | 4.55×10^-7^ | 4.59×10^-5^ | 1.74×10^-6^ | 7.69×10^-8^ |
| Ku80 | 4.41×10^-6^ | 3.43×10^-6^ | 6.24×10^-8^ | 1.21×10^-6^ | 8.01×10^-8^ | 3.08×10^-8^ |
| BRCA1 | 1.76×10^-6^ | 6.13×10^-7^ | 1.47×10^-8^ | 1.12×10^-6^ | 1.99×10^-7^ | 3.70×10^-9^ |
| BRCA2 | 5.43×10^-5^ | 5.36×10^-6^ | 4.75×10^-8^ | 6.50×10^-5^ | 1.35×10^-5^ | 9.52×10^-8^ |
| RAD51 | 0.18×10^-3^ | 1.59×10^-5^ | 3.65×10^-5^ | 0.21×10^-3^ | 1.13×10^-5^ | 3.85×10^-5^ |

**Table S5. Summary of P values for protein fold variation of combination of dual inhibitors with RT in relative to combination of single inhibitors with RT or RT alone in PC-3RR cells**

**Note**: N/A means “not applicable”.
