# Supplementary material for: PI3K/Akt/mTOR pathway inhibitors enhance radiosensitivity in radioresistant prostate cancer cells through inducing apoptosis, reducing autophagy, suppressing NHEJ and HR repair pathways
Source: Cell Death Dis. 2014 Oct 2;5(10):e1437–. doi: 10.1038/cddis.2014.415 (PMC4237243; doi:10.1038/cddis.2014.415)
Supplement: Supplementary Table S6 [file cddis2014415x6.doc]

|  | ***P* value** | | | | | |
| --- | --- | --- | --- | --- | --- | --- |
| **DU145**  **RR cell** | **BEZ235+RT VS BKM120+RT** | **BEZ235+RT VS Rapamycin+RT** | **BEZ235+RT VS**  **6 Gy RT** | **PI103+RT VS BKM120+RT** | **PI103+RT**  **VS Rapamycin+RT** | **PI103+RT VS**  **6 Gy RT** |
| p53 | 0.43 | 0.36 | 0.46 | 0.94 | 4.35 | 0.18 |
| P-p53 | 0.79×10^-3^ | 0.20×10^-2^ | 4.72×10^-5^ | 0.14×10^-2^ | 0.35×10^-2^ | 6.18×10^-5^ |
| p21 | 0.44×10^-3^ | 0.22×10^-1^ | 0.91×10^-2^ | 0.88×10^-3^ | 0.90×10^-2^ | 0.55×10^-2^ |
| CDK1 | 0.33 | 0.80×10^-1^ | 0.36×10^-1^ | 0.76×10^-1^ | 0.29 | 0.17×10^-1^ |
| P-CDK1 | 0.19×10^-2^ | 2.19×10^-5^ | 1.08×10^-6^ | 1.62×10^-5^ | 2.90×10^-6^ | 7.60×10^-8^ |
| Chk1 | 0.16 | 0.41 | 0.13 | 0.45×10^-1^ | 0.41×10^-1^ | 0.6×10^-1^ |
| P-Chk1 | 1.6×10^-5^ | 6.68×10^-7^ | 5.80×10^-8^ | 0.20×10^-2^ | 2.05×10^-5^ | 2.21×10^-6^ |
| Chk2 | 0.52 | 0.36 | 0.92 | 0.18 | 0.20 | 0.63 |
| P-Chk2 | 0.91×10^-3^ | 0.98×10^-3^ | 2.02×10^-5^ | 2.28×10^-5^ | 2.34×10^-5^ | 1.46×10^-7^ |
| Rb | 0.33 | 0.83×10^-1^ | 0.31 | 0.20 | 0.13 | 0.48×10^-1^ |
| P-Rb | 0.19×10^-2^ | 0.26×10^-2^ | 0.11×10^-3^ | 0.18×10^-3^ | 0.23×10^-3^ | 2.25×10^-5^ |
| active caspase-3 | 0.74×10^-3^ | 0.51×10^-3^ | 2.54×10^-5^ | 0.96×10^-3^ | 0.74×10^-3^ | 1.67×10^-5^ |
| active caspase-7 | 4.94×10^-5^ | 1.56×10^-5^ | 9.17×10^-5^ | 0.36×10^-3^ | 0.17×10^-3^ | 0.28×10^-3^ |
| cleaved PARP-1 | 0.93×10^-3^ | 0.41×10^-2^ | 0.15×10^-3^ | 0.46×10^-2^ | 0.17×10^-1^ | 0.42×10^-3^ |
| Bcl-2 | 0.12×10^-2^ | 0.59×10^-3^ | 0.36×10^-3^ | 3.01×10^-5^ | 7.49×10^-5^ | 7.45×10^-5^ |
| Bcl-xl | 0.51×10^-2^ | 0.39×10^-3^ | 0.14×10^-3^ | 0.16×10^-1^ | 2.34×10^-5^ | 8.89×10^-6^ |
| Bax | 0.24×10^-3^ | 0.13×10^-3^ | 3.66×10^-6^ | 2.50×10^-6^ | 1.41×10^-6^ | 6.41×10^-8^ |
| Beclin-1 | 0.14×10^-2^ | 0.97×10^-3^ | 0.77×10^-3^ | 8.76×10^-5^ | 9.25×10^-5^ | 7.15×10^-5^ |
| LC3A/B | 0.73×10^-2^ | 0.71×10^-2^ | 0.45×10^-3^ | 0.2×10^-1^ | 0.16×10^-1^ | 0.16×10^-2^ |
| γH2AX | 2.96×10^-6^ | 2.83×10^-5^ | 4.00×10^-6^ | 2.19×10^-5^ | 0.11×10^-3^ | 1.78×10^-5^ |
| Ku70 | 5.60×10^-5^ | 3.34×10^-5^ | 2.48×10^-5^ | 6.18×10^-5^ | 3.14×10^-6^ | 2.18×10^-5^ |
| Ku80 | 0.87×10^-3^ | 3.36×10^-5^ | 3.12×10^-5^ | 0.51×10^-2^ | 0.20×10^-3^ | 0.12×10^-3^ |
| BRCA1 | 0.14×10^-1^ | 0.66×10^-2^ | 1.81×10^-3^ | 0.33×10^-2^ | 0.94×10^-2^ | 1.59×10^-6^ |
| BRCA2 | 0.14×10^-2^ | 0.33×10^-3^ | 0.23×10^-3^ | 2.65×10^-5^ | 4.70×10^-6^ | 6.09×10^-6^ |
| RAD51 | 3.47×10^-5^ | 1.34×10^-5^ | 5.50×10^-5^ | 1.59×10^-5^ | 5.91×10^-6^ | 6.55×10^-5^ |

**Table S6.Summary of P values for protein fold variation of combination of dual inhibitors with RT in relative to combination of single inhibitors with RT or RT alone in DU145RR cells**
