# Supplementary material for: PI3K/Akt/mTOR pathway inhibitors enhance radiosensitivity in radioresistant prostate cancer cells through inducing apoptosis, reducing autophagy, suppressing NHEJ and HR repair pathways
Source: Cell Death Dis. 2014 Oct 2;5(10):e1437–. doi: 10.1038/cddis.2014.415 (PMC4237243; doi:10.1038/cddis.2014.415)
Supplement: Supplementary Table S7 [file cddis2014415x7.doc]

|  | ***P* value** | | | | | |
| --- | --- | --- | --- | --- | --- | --- |
| **LNCaP**  **RR cell** | **BEZ235+RT VS BKM120+RT** | **BEZ235+RT VS Rapamycin+RT** | **BEZ235+RT VS**  **6 Gy RT** | **PI103+RT VS BKM120+RT** | **PI103+RT**  **VS Rapamycin+RT** | **PI103+RT VS**  **6 Gy RT** |
| p53 | 0.84 | 0.062 | 0.098 | 0.39 | 0.13 | 0.67 |
| P-p53 | 6.03×10^-5^ | 6.58×10^-5^ | 1.74×10^-5^ | 0.41×10^-3^ | 0.96×10^-3^ | 6.77×10^-5^ |
| p21 | 0.87×10^-3^ | 0.11×10^-1^ | 0.13×10^-3^ | 0.14×10^-2^ | 0.13×10^-1^ | 0.15×10^-3^ |
| CDK1 | 0.58 | 0.31 | 0.93 | 0.90 | 0.082 | 0.18 |
| P-CDK1 | 4.51×10^-5^ | 5.34×10^-5^ | 2.03×10^-5^ | 0.13×10^-3^ | 0.14×10^-3^ | 3.84×10^-5^ |
| Chk1 | 0.39 | 0.043 | 0.041 | 0.48 | 0.55 | 0.048 |
| P-Chk1 | 0.79×10^-3^ | 1.54×10^-5^ | 7.33×10^-6^ | 0.41×10^-3^ | 4.18×10^-6^ | 2.58×10^-6^ |
| Chk2 | 0.059 | 0.94 | 0.26 | 0.34 | 0.063 | 0.27 |
| P-Chk2 | 3.32×10^-5^ | 4.08×10^-6^ | 9.67×10^-6^ | 0.46×10^-3^ | 7.10×10^-5^ | 7.83×10^-5^ |
| Rb | 0.084 | 0.24 | 0.28 | 0.42 | 0.32 | 0.87 |
| P-Rb | 3.80×10^-5^ | 4.53×10^-5^ | 8.31×10^-6^ | 0.18×10^-3^ | 0.16×10^-3^ | 4.51×10^-5^ |
| active caspase-3 | 0.88×10^-3^ | 6.19×10^-5^ | 0.74×10^-3^ | 0.22×10^-1^ | 0.26×10^-2^ | 0.32×10^-2^ |
| active caspase-7 | 0.15×10^-3^ | 0.42×10^-3^ | 1.67×10^-5^ | 9.15×10^-5^ | 0.30×10^-3^ | 8.71×10^-6^ |
| cleaved PARP-1 | 0.84×10^-2^ | 0.88×10^-2^ | 0.26×10^-3^ | 0.65×10^-2^ | 0.15×10^-1^ | 0.78×10^-3^ |
| Bcl-2 | 5.34×10^-5^ | 0.46×10^-3^ | 0.11×10^-3^ | 0.74×10^-2^ | 0.65×10^-2^ | 0.20×10^-2^ |
| Bcl-xl | 8.99×10^-6^ | 8.23×10^-7^ | 7.64×10^-8^ | 0.15×10^-3^ | 3.69×10^-5^ | 4.50×10^-6^ |
| Bax | 2.87×10^-5^ | 9.12×10^-6^ | 2.02×10^-6^ | 0.25×10^-3^ | 0.11×10^-3^ | 4.18×10^-5^ |
| Beclin-1 | 8.20×10^-5^ | 3.56×10^-5^ | 5.48×10^-6^ | 0.13×10^-1^ | 0.16×10^-1^ | 0.12×10^-2^ |
| LC3A/B | 0.17×10^-2^ | 2.84×10^-6^ | 1.44×10^-6^ | 0.19×10^-2^ | 2.85×10^-6^ | 5.53×10^-6^ |
| γH2AX | 0.60×10^-3^ | 0.38×10^-3^ | 0.13×10^-3^ | 0.52×10^-3^ | 0.42×10^-3^ | 0.14×10^-3^ |
| Ku70 | 0.48×10^-3^ | 1.79×10^-5^ | 2.66×10^-6^ | 0.47×10^-1^ | 0.24×10^-2^ | 0.12×10^-3^ |
| Ku80 | 0.12×10^-3^ | 1.95×10^-5^ | 4.13×10^-7^ | 0.43×10^-3^ | 4.23×10^-5^ | 1.79×10^-6^ |
| BRCA1 | 1.16×10^-5^ | 1.21×10^-5^ | 5.36×10^-7^ | 0.23×10^-3^ | 0.35×10^-3^ | 1.35×10^-5^ |
| BRCA2 | 2.28×10^-5^ | 9.81×10^-6^ | 3.51×10^-6^ | 0.61×10^-3^ | 0.12×10^-3^ | 6.84×10^-5^ |
| RAD51 | 1.70×10^-5^ | 0.37×10^-3^ | 1.35×10^-6^ | 0.10×10^-3^ | 0.48×10^-3^ | 1.42×10^-5^ |

**Table S7. Summary of P values for protein fold variation of combination of dual inhibitors with RT in relative to combination of single inhibitors with RT or RT alone in LNCaPRR cells**
