# Supplementary material for: PI3K/Akt/mTOR pathway inhibitors enhance radiosensitivity in radioresistant prostate cancer cells through inducing apoptosis, reducing autophagy, suppressing NHEJ and HR repair pathways
Source: Cell Death Dis. 2014 Oct 2;5(10):e1437–. doi: 10.1038/cddis.2014.415 (PMC4237243; doi:10.1038/cddis.2014.415)
Supplement: Supplementary Table S8 [file cddis2014415x8.doc]

| **Antibody** | **Source** | **Type** | **Dilution** | **Incubation time (min)** | **Temperature** |
| --- | --- | --- | --- | --- | --- |
| Rabbit anti-human Ki67 | Abcam | PAb | 1:1000 | O/N | 4℃ |
| Mouse anti-human p53 | Abcam | MAb | 1:1000 | O/N | 4℃ |
| Rabbit anti-human p53 (phospho S46) | Abcam | MAb | 1:1000 | O/N | 4℃ |
| Rabbit anti-human p21 | Abcam | PAb | 1:2000 | O/N | 4℃ |
| Rabbit anti-human CDK1 | Abcam | MAb | 1:1000 | O/N | 4℃ |
| Rabbit anti-human CDK1(phospho T14) | Abcam | PAb | 1:1000 | O/N | 4℃ |
| Rabbit anti-human Chk1 | Abcam | PAb | 1:500 | O/N | 4℃ |
| Rabbit anti-human Chk1 (phospho S345) | Abcam | PAb | 1:1000 | O/N | 4℃ |
| Rabbit anti-human Chk2 | Abcam | PAb | 1:500 | O/N | 4℃ |
| Rabbit anti-human Chk2 (phospho T68) | Abcam | PAb | 1:1000 | O/N | 4℃ |
| Rabbit anti-human Rb | Abcam | PAb | 1:1000 | O/N | 4℃ |
| Rabbit anti-human Phospho-Rb | Cell Signaling | PAb | 1:1000 | O/N | 4℃ |
| Rabbit anti-human Caspase-3 (Active) | Abcam | PAb | 1:500 | O/N | 4℃ |
| Rabbit anti-human Caspase-7 | Abcam | PAb | 1:2000 | O/N | 4℃ |
| Rabbit anti-human cleaved PARP | Abcam | MAb | 1:1000 | O/N | 4℃ |
| Mouse anti-human Bcl-2 | Abcam | MAb | 1:1000 | O/N | 4℃ |
| Rabbit anti-human Bcl-xl | Abcam | MAb | 1:1000 | O/N | 4℃ |
| Rabbit anti-human Bax | Abcam | MAb | 1:1000 | O/N | 4℃ |
| Rabbit anti-human Beclin-1 | Cell Signaling | PAb | 1:1000 | O/N | 4℃ |
| Rabbit anti-human LC3A/B | Abcam | PAb | 1:400 | O/N | 4℃ |
| Mouse anti-human γH2AX | Abcam | PAb | 1:1000 | O/N | 4℃ |
| Rabbit anti-human Ku70 | Abcam | MAb | 1:1000 | O/N | 4℃ |
| Rabbit anti-human Ku80 | Abcam | MAb | 1:1000 | O/N | 4℃ |
| Mouse anti-human BRCA1 | Abcam | MAb | 1:50 | O/N | 4℃ |
| Rabbit anti-human BRCA2 | Abcam | PAb | 1:1000 | O/N | 4℃ |
| Mouse anti-human Rad51 | Abcam | PAb | 1:1000 | O/N | 4℃ |
| Mouse anti-human β-tubulin | Sigma | MAb | 1:10000 | O/N | 4℃ |
| Mouse anti-human GAPDH | Merck Millipore | MAb | 1:600 | O/N | 4℃ |

**Table S8.** Antibodies used for western blot (WB)

**Abbreviations:** MAb: monoclonal antibody; O/N: overnight; PAb: polyclonal antibody; RT: room temperature
