# Supplementary material for: PI3K/Akt/mTOR pathway inhibitors enhance radiosensitivity in radioresistant prostate cancer cells through inducing apoptosis, reducing autophagy, suppressing NHEJ and HR repair pathways
Source: Cell Death Dis. 2014 Oct 2;5(10):e1437–. doi: 10.1038/cddis.2014.415 (PMC4237243; doi:10.1038/cddis.2014.415)
Supplement: Supplementary Table S9 [file cddis2014415x9.doc]

| **Inhibitor** | **Type of targeting protein(s)** | **Alias** | **Solvent** | **Final concentration** | **Source** |
| --- | --- | --- | --- | --- | --- |
| BEZ235 | PI3K and mTOR | NVP-BEZ235 | Chloroform | 1 mg/mL | Cayman Chemical |
| PI103 | PI3K and mTOR | N/A | Chloroform | 0.25 mg/mL | Cayman Chemical |
| BKM120 | PI3K | NVP-BKM120 /Buparlisib | DMSO | 82 mg/mL | Selleckchem |
| Rapamycin | mTOR | Sirolimus  /AY22989  /WY-090217 | DMSO | 20 mg/mL | Selleckchem |

**Table S9.** Summary of the information of dual and single PI3K/mTOR inhibitors
