# Supplementary figures and images for: PI3K/Akt/mTOR pathway inhibitors enhance radiosensitivity in radioresistant prostate cancer cells through inducing apoptosis, reducing autophagy, suppressing NHEJ and HR repair pathways
Source: Cell Death Dis. 2014 Oct 2;5(10):e1437–. doi: 10.1038/cddis.2014.415 (PMC4237243; doi:10.1038/cddis.2014.415)

## Slide 1
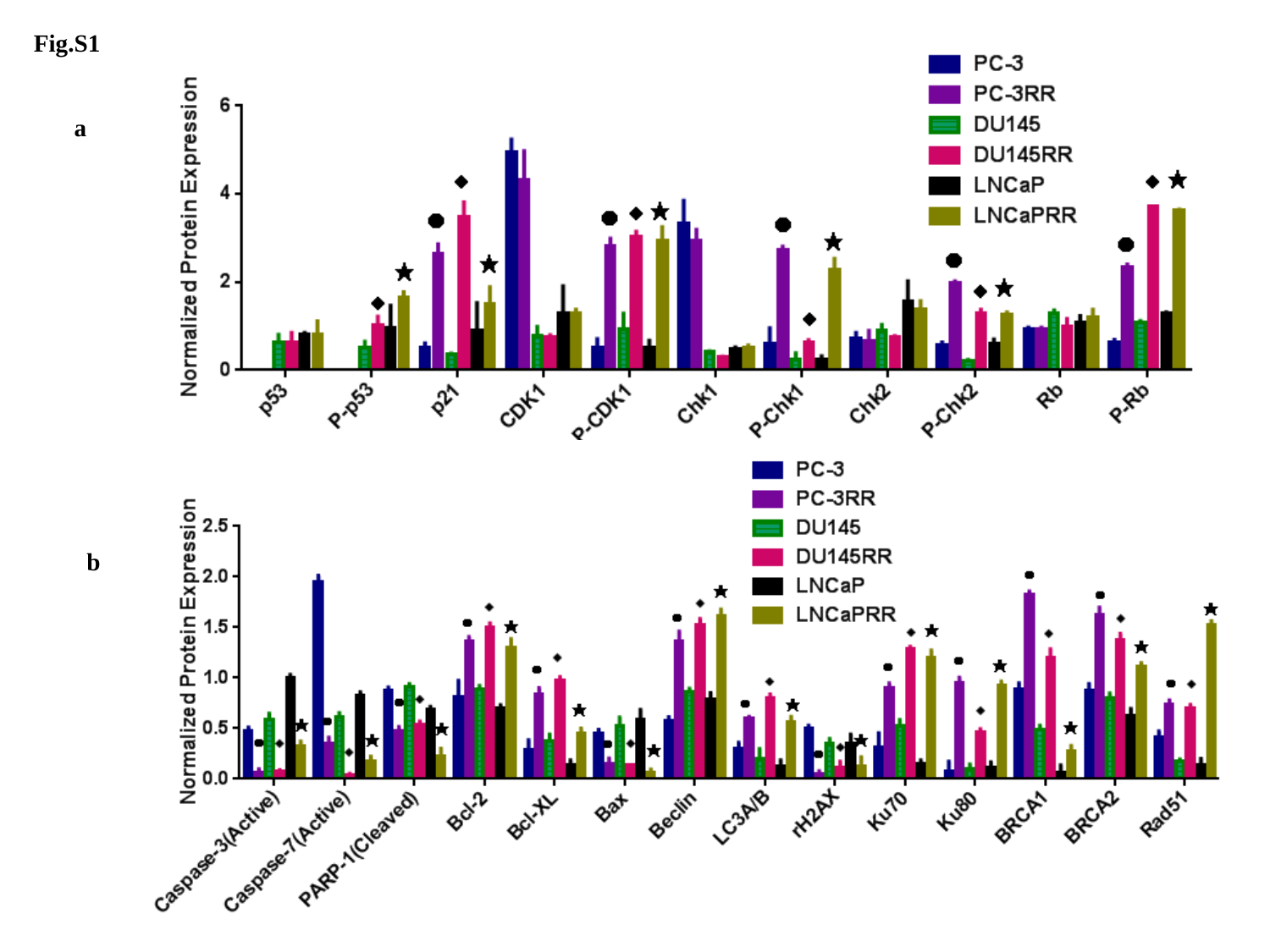

Fig.S1
a
b

Supplement: Supplementary Figure S1 [file cddis2014415x10.ppt]

## Slide 1
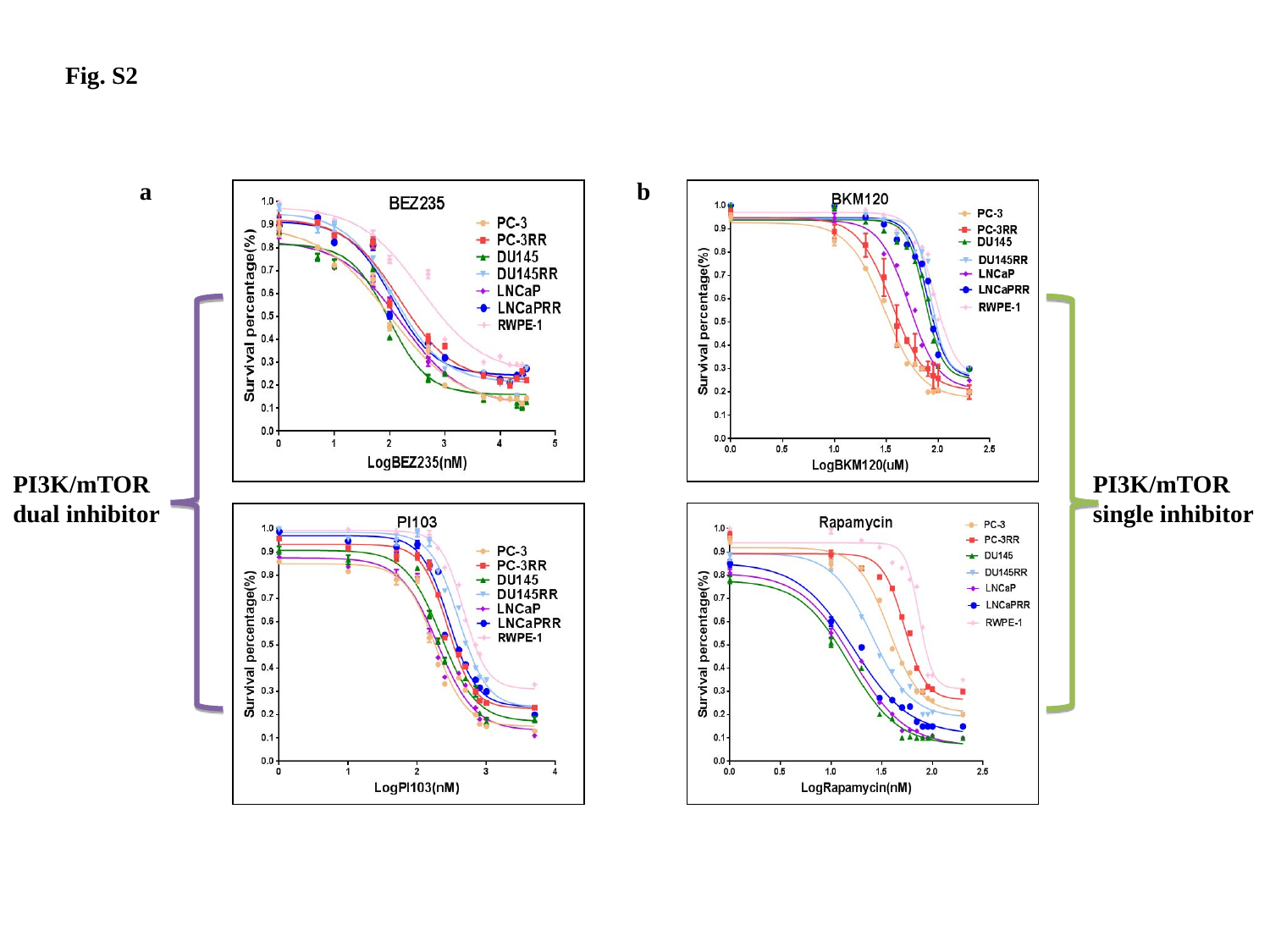

Fig. S2
a
b
PI3K/mTOR dual inhibitor
PI3K/mTOR single inhibitor

Supplement: Supplementary Figure S2 [file cddis2014415x11.ppt]

## Slide 1
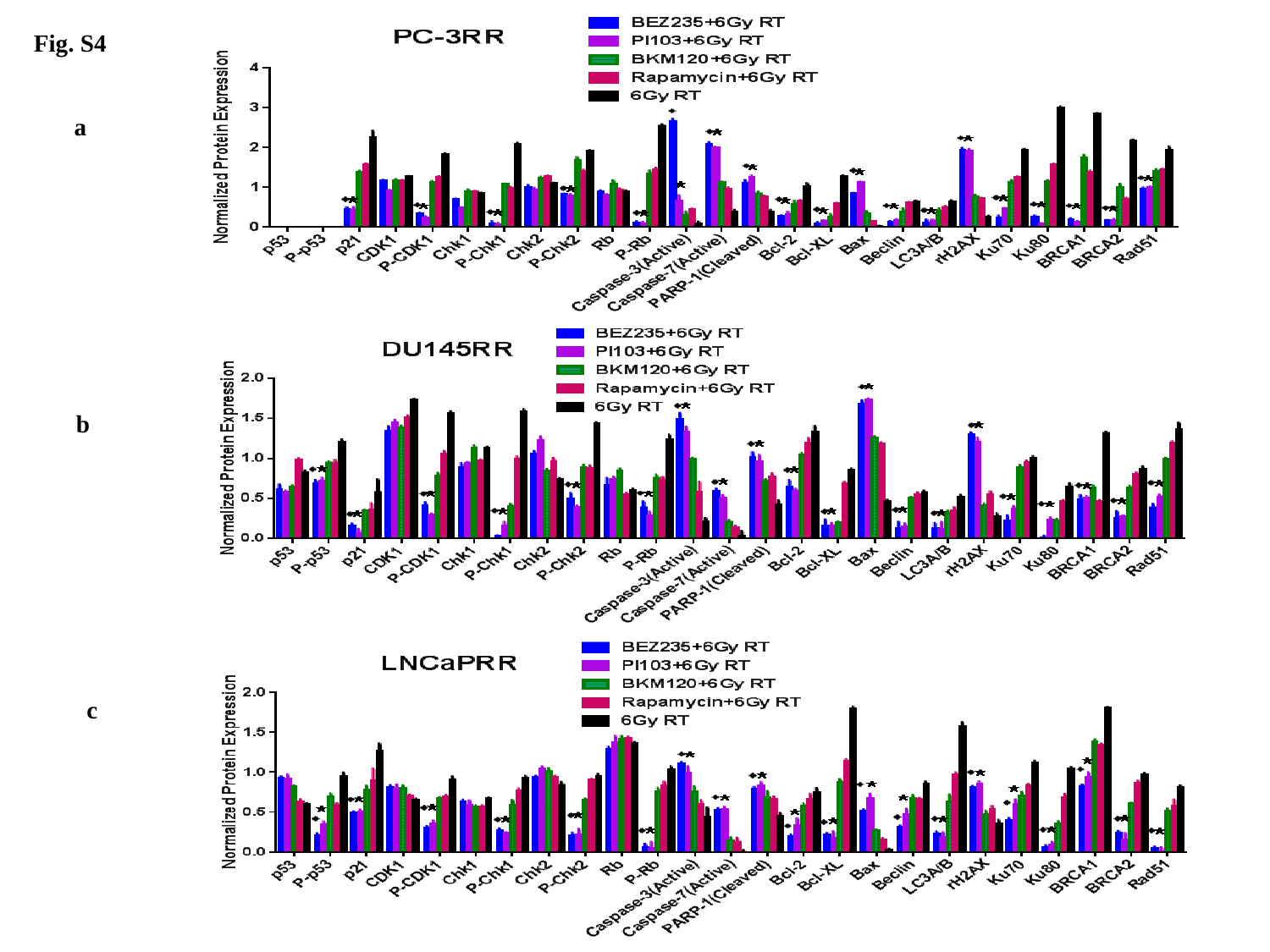

Fig. S4
a
b
c

Supplement: Supplementary Figure S4 [file cddis2014415x13.ppt]
