# Supplementary material for: PI3K/Akt/mTOR pathway inhibitors enhance radiosensitivity in radioresistant prostate cancer cells through inducing apoptosis, reducing autophagy, suppressing NHEJ and HR repair pathways
Source: Cell Death Dis. 2014 Oct 2;5(10):e1437–. doi: 10.1038/cddis.2014.415 (PMC4237243; doi:10.1038/cddis.2014.415)
Supplement: Supplementary Figure S3 [file cddis2014415x12.ppt]

## Slide 1
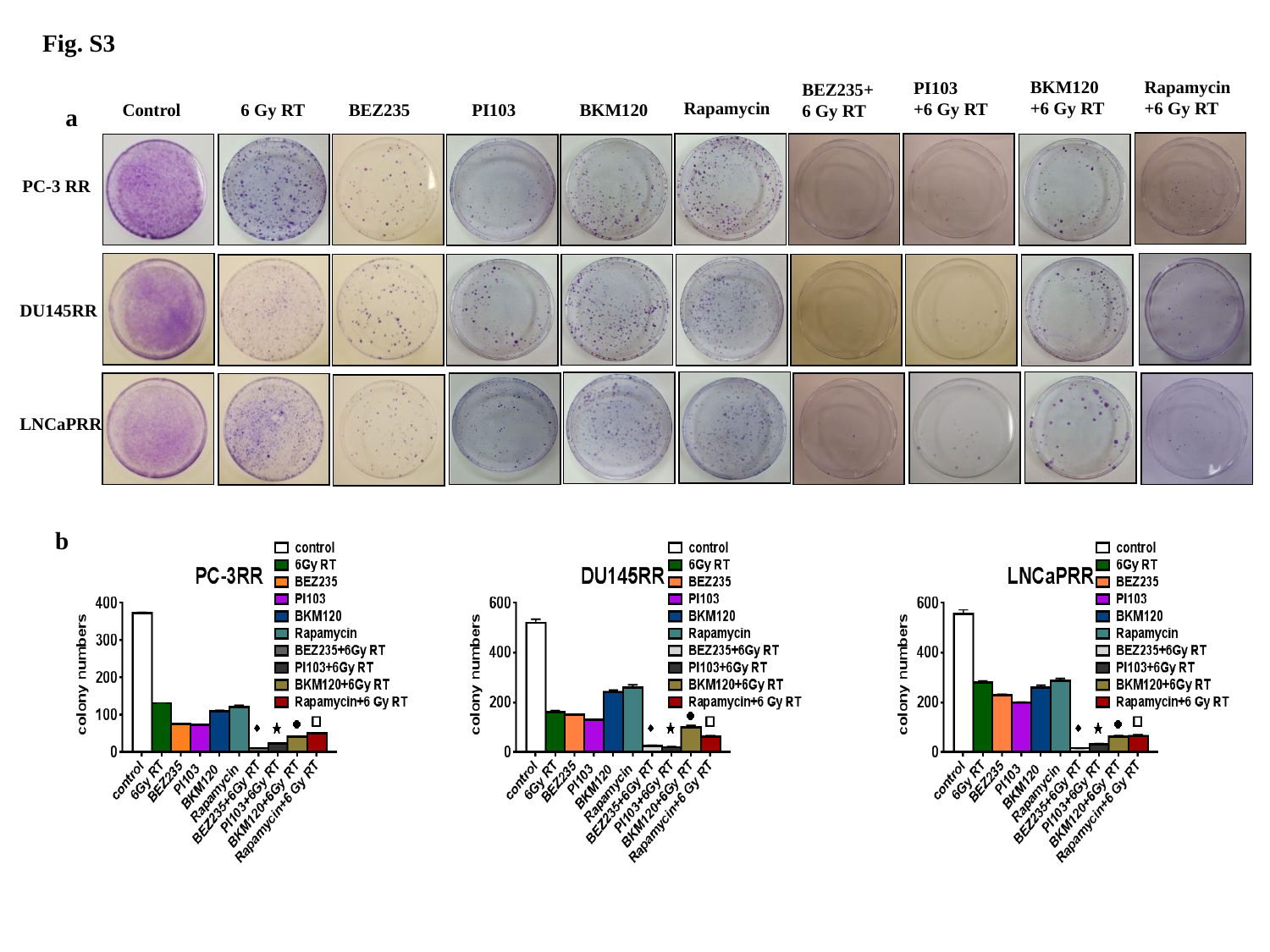

Fig. S3
BKM120
+6 Gy RT
Rapamycin
+6 Gy RT
PI103
+6 Gy RT
BEZ235+6 Gy RT
Rapamycin
Control
6 Gy RT
BEZ235
PI103
BKM120
PC-3 RR
DU145RR
LNCaPRR
a
b
