# Supplementary material for: PI3K/Akt/mTOR pathway inhibitors enhance radiosensitivity in radioresistant prostate cancer cells through inducing apoptosis, reducing autophagy, suppressing NHEJ and HR repair pathways
Source: Cell Death Dis. 2014 Oct 2;5(10):e1437–. doi: 10.1038/cddis.2014.415 (PMC4237243; doi:10.1038/cddis.2014.415)
Supplement: Supplementary Figure Legends [file cddis2014415x14.doc]

**Supplementary Figure Legends**

**Figure S1.** Quantification of Western blotting results from CaP-control and CaP-RR cells. (**a**)

The protein expression of p53, P-p53, p21, CDK1, P-CDK1, Chk1, P-Chk1, Chk2, P-Chk2, Rb and P-Rb in CaP-control and RR cell lines was normalized by the level of GAPDH. (**b**) The protein expression of active Caspase-3, active Caspase-7, cleaved PARP-1, Bcl-2, Bcl-xl, Bax, Beclin-1,LC3A/B, γH2AX, Ku70, Ku80, BRCA1, BRCA2 and RAD51 in CaP-control and RR cell lines was normalized by the level of GAPDH. Results are expressed as mean ± SD (n=3). ● indicates the difference between PC-3 and PC-3RR(*P*<0.05). ⬥ indicates the difference between DU145 and DU145RR(*P*<0.05). 🟉 indicates the difference between LNCaP and LNCaPRR(*P*<0.05).

**Figure S2.** Effects of PI3K/mTOR inhibitors by MTT assay. (**a**) Cell growth inhibition for two PI3K/mTOR dual inhibitors was assessed by MTT assay, respectively. (**b**) Cell growth inhibition for two PI3K/mTOR single inhibitors was assessed by MTT assay, respectively. Representative survival curves for dual and single inhibitors at 24 h are shown. The results were from three independent experiments (n=3).

**Figure S3**. Effects of combination treatment with inhibitors and RT, RT alone, or inhibitor alone on colony formation of CaP-RR cells. (**a**) Typical images of colony growth for the different treatments are shown. (**b**) Comparision of colony growth rates after different treatments. ⬥ indicates the difference between combination of BEZ235 with RT and BEZ235 alone in CaP-RR cells (*P*<0.05). 🟉 indicates the difference between combination of PI103 with RT and PI103 alone in CaP-RR cells (*P*<0.05). ● indicates the difference between combination of BKM120 with RT and BKM120 alone in CaP-RR cells (*P*<0.05). □ indicates the difference between combination of Rapamycin with RT and Rapamycin alone in CaP-RR cells (*P*<0.05). The results were from three independent experiments (n=3).

**Figure S4.** Quantification of Western blotting results from CaP-control and CaP-RR cells with different combination treatments. (**a**) The protein expression of p53, P-p53, p21, CDK1, P-CDK1, Chk1, P-Chk1, Chk2, P-Chk2, Rb, P-Rb, active Caspase-3, active Caspase-7, cleaved PARP-1, Bcl-2, Bcl-xl, Bax, Beclin-1,LC3A/B, γH2AX, Ku70, Ku80, BRCA1, BRCA2 and RAD51 in PC-3 and PC-3RR cell lines was normalized by the level of GAPDH. (**b**) The protein expression of p53, P-p53, p21, CDK1, P-CDK1, Chk1, P-Chk1, Chk2, P-Chk2, Rb, P-Rb, active Caspase-3, active Caspase-7, cleaved PARP-1, Bcl-2, Bcl-xl, Bax, Beclin-1,LC3A/B, γH2AX, Ku70, Ku80, BRCA1, BRCA2 and RAD51 in DU145 and DU145RR cell lines was normalized by the level of GAPDH. (**c**) The protein expression of p53, P-p53, p21, CDK1, P-CDK1, Chk1, P-Chk1, Chk2, P-Chk2, Rb, P-Rb, active Caspase-3, active Caspase-7, cleaved PARP-1, Bcl-2, Bcl-xl, Bax, Beclin-1,LC3A/B, γH2AX, Ku70, Ku80, BRCA1, BRCA2 and RAD51 in LNCaP and LNCaPRR cell lines was normalized by the level of GAPDH. ® indicates the difference between combination of BEZ235 with RT and combination of single inhibitor (BKM120 or Rapamycin) with RT or RT alone in CaP-RR cells (*P*<0.05). 🟉 indicates the difference between combination of PI103 with RT and combination of single inhibitor (BKM120 or Rapamycin) with RT or RT alone in CaP-RR cells (*P*<0.05). All results were from three independent experiments (n=3).
